# The role of Ca^2+^/Calcineurin/NFAT signalling pathway in osteoblastogenesis

**DOI:** 10.1111/cpr.13122

**Published:** 2021-09-15

**Authors:** Ranyue Ren, Jiachao Guo, Yangmengfan Chen, Yayun Zhang, Liangxi Chen, Wei Xiong

**Affiliations:** ^1^ Department of Orthopedics Tongji Hospital Tongji Medical College Huazhong University of Science and Technology Wuhan Hubei China; ^2^ Department of Pediatric Surgery Tongji Hospital Tongji Medical College Huazhong University of Science and Technology Wuhan Hubei China; ^3^ Department of Trauma and Reconstructive Surgery Siegfried Weller Research Institute BG Trauma Center Tübingen University of Tübingen Tübingen Germany

**Keywords:** Ca^2+^, calcineurin, calmodulin, NFAT, osteoblastogenesis

## Abstract

The bone remodelling process is closely related to bone health. Osteoblasts and osteoclasts participate in the bone remodelling process under the regulation of various factors inside and outside. Excessive activation of osteoclasts or lack of function of osteoblasts will cause occurrence and development of multiple bone‐related diseases. Ca^2+^/Calcineurin/NFAT signalling pathway regulates the growth and development of many types of cells, such as cardiomyocyte differentiation, angiogenesis, chondrogenesis, myogenesis, bone development and regeneration, etc. Some evidences indicate that this signalling pathway plays an extremely important role in bone formation and bone pathophysiologic changes. This review discusses the role of Ca^2+^/Calcineurin/NFAT signalling pathway in the process of osteogenic differentiation, as well as the influence of regulating each component in this signalling pathway on the differentiation and function of osteoblasts, whereby the relationship between Ca^2+^/Calcineurin/NFAT signalling pathway and osteoblastogenesis could be deeper understood.

## Graphical abstract

Non‐classical Wnt signalling pathway, Orai1/STIM1, L‐VGCC and NCX can regulate the activity of CaM and CaN by modulating the concentration of Ca^2+^ in osteogenic precursor cells, thereby affecting the nuclear metastasis of NFAT and the process of osteogenic differentiation.

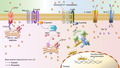

## 1 INTRODUCTION

Skeletal system is continuously in the process of dynamic self‐renewal, during that old and damage bone is removed and new bone is generated, this process is also called ‘bone remodelling’. The bone remodelling process will last a lifetime to prevent the accumulation of micro‐damages in bone. Osteoclast‐led bone resorption and osteoblast‐led bone formation are tightly coupled in bone remodelling under various physiological and pathological conditions and can be affected by a variety of inside and outside factors such as hormones, cytokines, mechanical forces, magnetic fields, etc.^1^ Excessive activation or lack of function of osteoblasts and osteoclasts is involved in the occurrence and development of various bone‐related diseases, such as osteoporosis, osteopetrosis, periodontitis, rheumatoid arthritis, rickets, tumours bone metastases, ankylosing spondylitis and Paget's disease.^2^, ^3^, ^4^, ^5^

Many signal cascades in cells are activated by the increase of Ca^2+^ concentration, including calcineurin/nuclear factor of activated T cell (CaN/NFAT),^6^ The Ca^2+^/calcineurin/nuclear factor of activated T‐cells (Ca^2+^/CaN/NFAT) signalling pathway was originally discovered in T cells,^7^, ^8^ which regulates the initiation of T‐cell immune responses and genes expression of immune‐related cytokines.^7^, ^8^ In subsequent studies, it was found that the Ca^2+^/CaN/NFAT signalling pathway plays a more significant role in the regulation of cell growth and development, such as regulating cardiomyocyte differentiation, chondrocyte differentiation, myocyte hypertrophy, angiogenesis, myogenesis, skeletal development and regeneration, etc.^8^, ^9^, ^10^, ^11^, ^12^, ^13^, ^14^, ^15^ Studies have shown that the (Down syndrome critical region 1) DSCR1 gene on chromosome 21 of patients with Down syndrome is overexpressed. DSCR1 gene is highly expressed in myocardium, striated muscle, neuronal cells and T cells, etc., the peptide expressed by this gene regulates CaN through competing with CaN to inhibit the CaN signalling pathway.^16^, ^17^ The symptom of skeletal dysplasia in Down syndrome patients is thought to be relevant to the overexpression of the DSCR1 gene.^18^ Sun et al.^19^ discovered that the level of bone formation of mice lacking the CaN Aα subtype was observably reduced and the mice showed osteoporosis. In addition, some studies reported that the activation of CaN was detected in the first batch of bone cells developed at the foetal stage^20^; acute and rapid bone loss occurred after organ transplantation patients were treated with CaN inhibitors^19^; mice and rats were treated with equivalent doses of calcineurin inhibitors, and increased bone resorption and bone loss could also be observed.^21^, ^22^, ^23^ A large number of studies have shown that Ca^2+^/CaN/NFAT signalling pathway plays an extremely important role in affecting bone resorption, bone formation and bone physiopathological changes. Unlike the role of Ca^2+^/CaN/NFAT signalling pathway in osteoclast differentiation and bone resorption, which has been thoroughly discussed, the influence of this signalling pathway on osteoblast differentiation still needs to be thoroughly summarized and analysed, besides, the conclusions reached by some studies are also contradictory. Therefore, this review summarizes, analyses and discusses the recent studies on the role of Ca^2+^/CaN/NFAT signalling pathway in osteoblast biology, and summarized the different effects of a variety of compounds that have a regulatory effect on the Ca^2+^/CaN/NFAT signalling pathway in osteogenic differentiation.

## 2 OSTEOBLASTOGENESIS AND RELATED SIGNALLING PATHWAYS

Osteoblasts are mainly derived from bone marrow mesenchymal stromal cells (BMSCs). BMSCs have the ability to differentiate into osteoblasts, adipocytes and chondrocytes, many transcription factors participate in the process of inducing BMSCs to differentiate into osteoblasts, such as runt‐related transcription factor 2 (RUNX2), β‐catenin and osteoblast‐specific transcription factor (Osterix), etc.^24^ After BMSCs are induced to differentiate into osteoblasts, they can secrete an uncalcified bone precursor composed of type I collagen, which is osteoid.^25^ Subsequently, mature osteoblasts secrete vesicles, and the alkaline phosphatase (ALP) in the vesicles combines with calcium ions to form hydroxyapatite, thereby osteoid calcification is achieved. The cytoplasm of osteoblasts embedded in the osteoid reduces and the osteoblasts then transform into osteocytes.^26^ In addition to the bone formation function, osteoblasts can also secrete a variety of cytokines and meet the needs of various physiological and pathological changes in autocrine and paracrine manners.^2^, ^27^

**FIGURE 1:**
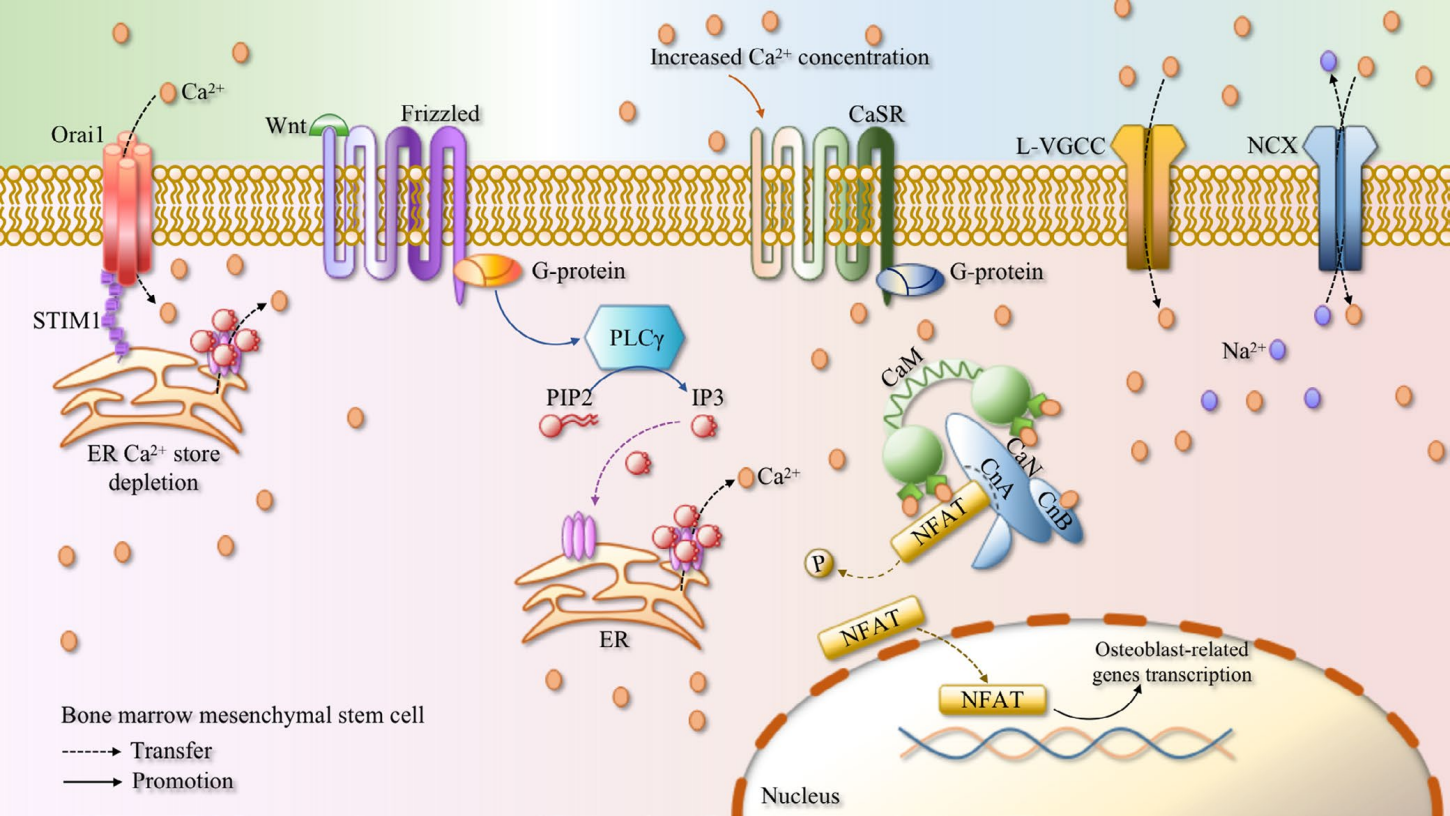
Concise schematic diagram of Ca^2+^/CaN/NFAT signalling pathway involved in the process of osteoblast differentiation. The combination of Wnt and the N‐terminal of Frizzled causes the activation of PLCγ and increases the level of IP3, which in turn activates the IP3 receptor to promote the release of Ca^2+^ from ER into the cytoplasm, and activates CaN by CaM to promote the nuclear translocation of NFAT. CaSR activates PLCγ in response to an increase in the extracellular Ca^2+^ concentration to cause an elevation in the intracellular Ca^2+^ concentration. In addition, the storage depletion of Ca^2+^ in ER can also lead to Ca^2+^ influx through Orai1/STIM1. L‐VGCC and NCX all regulate Ca^2+^ influx, which further results in the activation of the Ca^2+^/CaN/NFAT signalling pathway. NFAT and Osterix form transcription complexes, which in turn, trigger the transcription of osteoblast‐related genes

The differentiation of BMSCs into osteoblasts is regulated by a variety of signalling pathways, such as wingless‐type MMTV integration site (Wnt), transforming growth factor‐β/bone morphogenetic protein (TGF‐β/BMP), Hedgehogs and fibroblasts growth factor (FGF) signalling pathways.^28^ TGF‐β/BMP increase the expression of RUNX2 by activating Smad and mitogen‐activated protein kinase (MAPK) signalling pathways.^29^, ^30^, ^31^ The active fragments of Hedgehogs can bind to the G protein‐coupled receptor Smoothened (Smo), and also caused the increase of RUNX2 expression level by activating Smad.^28^, ^32^ FGF binds to its receptor to cause receptor dimerization, and promotes osteogenic differentiation by activating its downstream signalling pathways such as MAPK, JNK, PKC and PI3K.^33^ In BMSCs, Wnt protein transmits signals through canonical and non‐canonical pathways.^34^ The canonical Wnt signalling pathway is mediated by β‐catenin. Under unstimulated condition, β‐catenin in the cytoplasm is phosphorylated by the complex of glycogen synthase kinase‐3β (GSK‐3β), adenomatous polyposis coli (APC) and Axin, and together form a degradation complex. The complex will be further ubiquitinated and degraded by the proteasome system. Wnt protein binds to Frizzled and low‐density lipoprotein receptor related protein 5/6 (LRP5/6) receptors complex, causing inhibition of GSK‐3β activity, allowing β‐catenin to be released as a monomer and accumulate in the cytoplasm.^35^, ^36^ Then β‐catenin translocates to the nucleus and induces the expression of its target genes such as RUNX2 and PPARγ.^24^ The non‐canonical Wnt signalling pathway also plays an important role in the recruitment, maintenance and differentiation of BMSCs, the Ca^2+^/CaN/NFAT pathway has been shown to be activated by the non‐canonical Wnt signalling pathway during the differentiation of BMSCs into osteoblasts.^37^, ^38^, ^39^ The secreted glycoprotein Wnt functions in the form of autocrine or paracrine. Frizzled on the cell membrane belongs to the G protein‐coupled receptor, its N‐terminal can bind to the Wnt protein, and then cause the activation of PLCγ, activated PLCγ increases inositol 1,4,5‐triphosphate (IP3) level, and then promotes the release of Ca^2+^ from ER into the cytoplasm by activating the IP3 receptor, and activates CaN by activating CaM to promote the nuclear translocation of NFAT.^37^, ^40^ BMSCs also express calcium‐sensing receptor (CaSR), which activates PLCγ in response to increase in extracellular Ca^2+^ concentration, thereby producing IP3, promoting the release of Ca^2+^ from ER and causing the increase in intracellular Ca^2+^ concentration.^41^ Stromal interaction molecule 1 (STIM1) can sense the changes in the concentration of Ca^2+^ in ER. When Ca^2+^ in ER is depleted, STIM1 aggregates and interact with the Orai1 protein on the cell membrane to open the store‐operated Ca^2+^ (SOC) channel and accelerate Ca^2+^ influx,^42^, ^43^ which further contributes to the activation of CaN and NFAT nuclear translocation. NFAT and Osterix form transcriptional complexes in the nucleus, which subsequently trigger bone morphogenetic protein‐2 (BMP‐2),^44^ alpha‐1 type I collagen (ColIα1),^45^ osteopontin (OPN), ALP, osteocalcin (OCN) and other osteogenic‐related genes transcription and then promote osteogenic differentiation.^46^ Figure 1 exhibits the process of osteoblasts differentiation, which Ca^2+^/CaN/NFAT signalling pathway involved in.

## 3 CA^2+^/CAN/NFAT SIGNALLING PATHWAY IN OSTEOBLASTOGENESIS

### 3.1 Ca^2+^/CaN/NFAT signalling pathway

**FIGURE 2:**
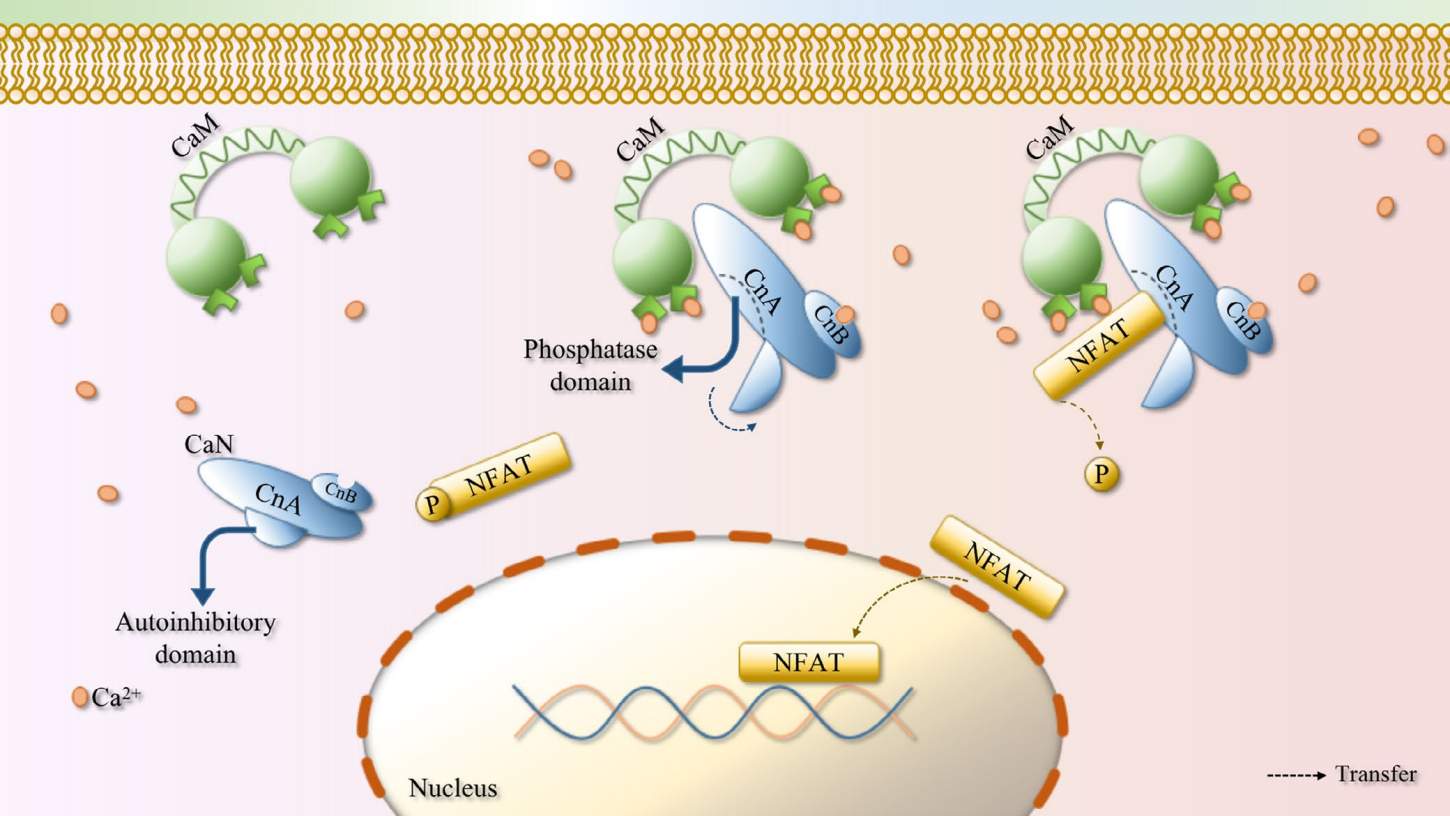
Brief schematic diagram of Ca2+/CaN/NFAT signalling pathway. Each globular domain of CaM has a pair of Ca^2+^ binding sequences, CaM exposes a hydrophobic surface after binding Ca^2+^. CaN is composed of catalytic subunit (CnA) and regulatory subunit (CnB). CnB is capable to bind Ca^2+^, CnA contains phosphatase domain (catalytic domain), CnB binding domain, CaM binding domain and autoinhibitory domain, the CaM binding domain can be combined with the hydrophobic surface of CaM and be regulated by it. In the resting state, autoinhibitory domain covers the phosphatase domain, the binding of Ca^2+^ with CnB and CaM/CaM with CnA make autoinhibitory domain separate itself from phosphatase domain, and CaN is then activated. Activated CaN dephosphorylates the serine residues of the NFAT regulatory domain, exposing the nuclear localization sequence, thereby promoting its transfer from the cytoplasm to the nucleus, and acting as a transcription factor in the nucleus to further cause the transcription of NFAT‐dependent genes

Increased intracellular concentration of Ca^2+^ can activate CaN by interacting with calmodulin (CaM).^47^ CaM is dumbbell‐shaped, its C‐terminus and N‐terminus each contain a globular domain, and the two globular domains are connected by a flexible helical joint region. Each globular domain of CaM has a pair of Ca^2+^ binding sequences, and after binding Ca^2+^, a hydrophobic surface is exposed.^15^ This hydrophobic surface can bind to a variety of CaM target proteins, including CaN. CaN is widely expressed in brain, lung, skeletal muscle, heart valve, myocardium, kidney, spleen, bone and other tissues,^48^, ^49^, ^50^ it is a type of serine/threonine phosphatase, and is a heterodimer, which is structurally composed of catalytic subunits (CnA) and regulatory subunit (CnB).^51^, ^52^ CnB possesses Ca^2+^ binding ability, CnA contains multiple domains, the more important of which are the phosphatase domain (catalytic domain), CnB binding domain, CaM binding domain and the self‐inhibitory domain. The CaM binding domain can combine with the hydrophobic surface of CaM, and then be regulated by CaM.^1^ Under static state, the self‐inhibition zone covers the phosphatase domain. After Ca^2+^ binding CnB and CaM/CaM binding CnA, the conformation of CaN alters, the inhibitory zone separates itself from the phosphatase domain, causing CaN to be activated.^53^ Activated CaN can dephosphorylate multiple substrates, including NFAT.^44^ NFAT contains a few domains, the regulatory domain of which are highly phosphorylated under the inactive state, which covers the nuclear localization sequence and makes the NFAT protein to remain in the cytoplasm.^51^ Activated CaN dephosphorylates the serine residues of NFAT regulatory domain, and changes the conformation of NFAT protein, exposing the nuclear localization sequence, which promotes its transfer from the cytoplasm to the nucleus, then NFAT acts as a transcription factor in the nucleus to further cause NFAT‐dependent genes transcription.^9^, ^54^, ^55^ NFAT1‐4 in the NFAT gene family are regulated by CaN and play an irreplaceable role in a variety of biological processes.^8^, ^56^ Figure 2 shows a schematic diagram of the Ca^2+^/CaN/NFAT signalling pathway.

### 3.2 Ca^2+^ in osteoblastogenesis

Ca^2+^ participates in a variety of signal transduction processes in cells. It can act as a secondary messenger or as a result of ion channel activation, affecting a variety of cell activities.^57^ Ca^2+^ released during bone resorption will increase the local concentration of extracellular Ca^2+^, which functions as a coupling factor between osteoclasts and osteoblasts to chemoattractant the migration of osteoblasts through CaSR.^58^, ^59^, ^60^, ^61^ Ca^2+^ plays an important role in the process of osteogenic differentiation. During dentinogenesis, it involves the influx of extracellular Ca^2+^ and the release of Ca^2+^ from the intracellular Ca^2+^ storage.^62^

L‐type voltage gated Ca^2+^ channel (L‐VGCC) participates in the proliferation of human BMSCs, MC3T3‐E1 osteoblasts and human periodontal ligament cells (hPDLCs), and mediates extracellular Ca^2+^ induced BMP‐2 signalling pathway activation and mineralization.^63^, ^64^, ^65^ It exhibits L‐VGCC dependence in the process of osteogenic differentiation of hPDLCs induced by Ca^2+^, and the inhibitor of L‐VGCC nifedipine inhibits its osteogenic differentiation.^65^

The Na^+^/Ca^2+^ exchanger (NCX) mediates Ca^2+^ flowing into the cells mainly through reverse exchange,^66^ and plays its role on the surface of osteoblasts, regulates the concentration of Ca^2+^ in osteoblasts and promote bone matrix mineralization.^57^, ^67^

The CaSR belonging to the G protein‐coupled receptors can sense the extracellular Ca^2+^ concentration and instantaneously mobilize intracellular Ca^2+^ flux.^68^ Previous studies have shown that CaSR signals mediate the osteogenic differentiation of BMSCs in vitro and bone formation in vivo, and CaSR agonists can promote the proliferation, differentiation and matrix mineralization of osteoblasts.^41^, ^69^, ^70^ In addition, Ca^2+^ influx caused by mechanically sensitive channels also promotes osteogenic differentiation^71^; Orai1 gene knockout leads to impaired osteoblast differentiation and mineralization.^43^

Overexpression of Pannexin 3, which is the Ca^2+^ channel of ER, can increase the intracellular Ca^2+^ concentration to promote osteogenic differentiation,^72^ and the depletion of Ca^2+^ in the ER induced by IP3 causes STIM1 to accumulate at the junction of the ER and cell membrane, STIM1 interacts with Orai1 protein and activates the SOC channel, causing Ca^2+^ influx,^73^ this process has also been demonstrated to take a part in dental pulp cells (DPCs) osteogenic differentiation and mineralization.^57^, ^74^

In addition, in the process of Ca^2+^ regulating osteogenic differentiation, there are also crosstalks between various Ca^2+^ channels. For example, Ca^2+^ can affect the proliferation and differentiation of osteoblasts through the mutual adjustment between CaSR‐L‐VGCC and SOC channel.^57^

When supplemented with Ca^2+^ (1.8–7.8 mM) in the culture medium of BMSCs, the cells exhibited larger area and circumference, as well as enhanced proliferation ability.^64^ Extracellular 10–15 mM Ca^2+^ stimulation can cause the activation of downstream MAPK signalling pathways through Ca^2+^ influx, thereby promoting the expression of osteogenic differentiation‐related genes such as FGF‐2, BMP‐2, OPN, OCN and RUNX2.^75^, ^76^, ^77^ The study by An et al. revealed that higher concentrations (5.4–16.2 mM) of Ca^2+^ did not affect the proliferation of DPCs, and did increase the mRNA levels of OCN and OPN, and enhanced their mineralization ability, but the mRNA levels of ALP and COL1A2 in cells decreased, ALP activity was also inhibited.^78^ When the extracellular Ca^2+^ concentration increases to 50 mM, it will hinder the normal adhesion of cells.^79^ Therefore, proper concentration of Ca^2+^ treatment can enhance osteogenic differentiation and mineralization, but excessively high concentrations of Ca^2+^ may disrupt Ca^2+^ homeostasis and cause abnormal cell function.

### 3.3 Calmodulin in osteoblastogenesis

Calmodulin is regulated by intracellular Ca^2+^ and activates a variety of downstream target proteins after binding Ca^2+^. It is precisely because there are many types of CaM downstream target proteins, the cell functions that CaM participates in are also diverse, such as inflammation, metabolism, apoptosis and so on.^80^ Conversely, CaM can also affect intracellular Ca^2+^ flux by regulating Ca^2+^ channels, such as through IP3R and P/Q type calcium channel.^15^ CaM participates in the process of parathyroid hormone (PTH) and vitamin D3 in regulating osteoblast differentiation through Ca^2+^ signals,^81^ and Smad1 in the BMP signalling pathway can directly bind to CaM, so that the activity of Smad1 is increased, thereby promoting osteogenic differentiation.^82^ Trifluoperazine, a CaM inhibitor, is demonstrated to inhibit the osteogenic differentiation of MC3T3‐E1 cells and bone formation in mice, and has a dose‐dependent inhibitory effect on the activity of ALP in rat skull.^83^, ^84^

### 3.4 Calcineurin in osteoblastogenesis

The calcium‐sensitive protein CaM can activate calcineurin under the condition of low and continuously increasing intracellular Ca^2+^ concentration.^85^ The activation of CaN can affect a variety of physiological and pathological processes by dephosphorylating various downstream proteins, such as T cell activation, vesicle transport, cell growth and apoptosis and so on. Sun et al. considered that CaN stimulated osteoclast differentiation, whilst inhibiting its bone resorption function, they reported that CaN‐deficient mice showed reduced osteoclastogenesis and increased osteoclast bone resorption activity, these two effects offset the changes in bone mass in mice. They also observed decreased osteoblast differentiation and severe osteoporosis in mice lacking the CaN catalytic subunit, and after TAT sequence was used to introduce CnA into mouse embryonic osteoblast precursor cells (MC3T3‐E1 cells), the expression of osteogenic marker genes RUNX2, ALP, bone sialoprotein (BSP) and OCN increased significantly, so Sun et al.^50^ concluded that osteoporosis in CaN‐deficient mice is caused by defects in bone formation. However, Yeo et al.^1^ disagree with the above views, they believed that the regulation of CaN signalling pathway in mice not only affected the differentiation of BMSCs into osteoblasts but also affected the physiological status of endothelial progenitor cells, immune cells, chondrocytes and adipocytes, and all these changes may interfere with osteogenic differentiation. Therefore, Yeo et al.^1^ constructed mice model that lacked the CaN regulatory subunit only in osteoblasts, and noticed that the levels of ALP, OCN, and collagen I (Coll I) in vivo rose, osteogenic differentiation degree elevated and bone mass increased. Yeo et al. claimed that low concentrations of cyclosporin A (CsA) (less than 1 μM in vitro, less than 35.5 nM in vivo) could increase the expression of Fos‐related antigen‐2 (Fra‐2), and Fra‐2 acted as a transcription factor to promote OCN and alpha‐2 type I collagen (ColIα2) transcription, thereby promoting osteogenic differentiation and bone formation.^86^, ^87^ However, high concentrations of CsA (more than 1 μM in vitro and in vivo) inhibited osteogenic differentiation and bone formation.^86^ Similar to the effect of CsA, low concentrations of FK506 (less than1 μM in vitro) promoted osteogenic differentiation,^88^ whilst even in the presence of BMP‐2, high concentrations of FK506 could reduce the expression of ColIα1 and BSP and other osteogenic‐related genes in vivo and in vitro, then inhibited osteogenic differentiation, and this effect was thought to be exerted by inhibiting the formation of the NFAT‐Osterix‐DNA complex.^45^ Sun et al. and Yeo et al., respectively, studied two different subunits of CaN and came to diametrically opposite conclusions. Amongst them, the loss or gain experiments of CnA function in the study of Sun et al. is systemic, whilst the research of Yeo et al. is limited to regulate CnB in mice osteoblasts. Therefore, specifically knockdown or overexpression of CnA in osteoblasts in vivo is necessary, so that it can further analyse its specific influence on osteoblast differentiation. Moreover, when investigating the influence of CaN inhibitors on osteogenic differentiation, researchers used a wide range of CaN inhibitors (from 1 nM to 25 μM) and agreed that high concentrations of CaN inhibitors suppressed osteogenic differentiation, and low concentrations of CaN inhibitors accelerated osteogenic differentiation, but this concentration range of the CaN inhibitor coincides with their concentration that induces osteoblast apoptosis,^89^ and it is reported that Endothelin‐1 (ET‐1) activated CaN signalling pathway when acting as an anti‐apoptotic factor for osteoblasts.^90^ Therefore, the exact conclusions and specific mechanisms of CaN inhibitors regulating osteoblast differentiation need to be further studied. It is worth noting that many studies have also mentioned the influence of osteoblast function when they reported that osteoblast differentiation is regulated by CaN signalling pathway, and they all claimed that the impacts on osteoblast function are the same as those on osteoblast differentiation, but they did not first culture mature osteoblasts and then regulate CaN signalling pathway, instead, they directly analysed the changes in osteoblast function through the mineralization level of osteoblasts whose differentiation degree has been altered. Therefore, the conclusions about the regulation of osteoblast function by CaN signalling pathway is not precise.

### 3.5 Nuclear factor of activated T cell in osteoblastogenesis

In the inactive state, NFAT protein localizes in the cytoplasm due to the hyperphosphorylation of its N‐terminal regulatory domain. After Ca^2+^ activates CaN through CaM, CaN dephosphorylates NFAT and exposes the nuclear localization sequence to cause its nuclear translocation.^91^ In the nucleus, NFAT acts as a transcription factor to promote the transcription of target genes and NFATc1 itself. It can be inferred that in the Ca^2+^/CaN/NFAT pathway, CaN not only regulates the dephosphorylation and nuclear translocation of NFAT but also enhances its expression; therefore, NFAT as a transcription factor can cause its own self‐amplification effect.^18^ Some previous studies reported that CaN/NFAT had a positive regulatory effect on osteogenic differentiation, and confirmed that the overexpression of NFAT in vivo and in vitro could promote osteogenic differentiation,^45^ after expressing the constitutively active variant of NFATc1 in osteoblasts in mice, the mice showed increased bone mass.^92^ Similarly, mice lacking NFAT had reduced bone formation and low bone mass,^45^ the inactivation of NFATc1 and NFATc2 markedly inhibited the differentiation and function of osteoblasts.^93^

Besides, the promoter of the Fra‐2 gene contains three potential NFAT consensus sequences, and the combination of NFAT with Fra‐2 will cause the negative regulation of Fra‐2,^86^ Yeo et al. found NFATc1 silencing increased the expression of Fra‐2, then promoted OCN and ColIα2 transcription, and accelerated osteoblastogenesis and bone formation.^86^, ^87^ Similar to this conclusion, Choo et al.^94^ found that the activity of ALP in the osteoblast cell line expressing constitutively active NFATc1 was inhibited, and the protein levels of Osterix and OCN were also reduced. Other studies have exhibited that in the SaOS‐2 human osteosarcoma cell line, NFATc1 inhibits bone formation by negatively regulating oestrogen receptor α (ERα).^95^ At present, there are still disagreements on the role of NFAT in the process of osteogenic differentiation, but these studies utilized different treatment methods for NFAT. In in vivo experiments, the constitutive expression or knockout of NFAT in some studies is not limited in the osteoblasts, but systemic. It is known that NFAT regulates a variety of physiological and pathological processes of cells, amongst them, the immune response can also have a certain effect on bone formation. Therefore, these conclusions may be not that accurate. However, the contrary conclusions drawn from the overexpression or knockout of NFAT in in vitro experiments still need to be further verified, and it is also necessary to determine whether it is affected by different types of osteoblast precursor cells, which are used and different transfection methods.

## 4 DIVERSE CA^2+^/CAN/NFAT SIGNALLING PATHWAY MODULATING COMPOUNDS, WHICH REGULATE OSTEOGENIC DIFFERENTIATION

Decreased differentiation or dysfunction of osteoblasts will lead to a variety of skeletal diseases. The Ca^2+^/CaN/NFAT signalling pathway has been shown to be closely related with the physiological activities of osteoblasts. We have summarized compounds that have a regulatory effect on this signalling pathway and at the same time modulate osteoblastogenesis, aiming to provide new ideas for the exploration of treatment options for osteogenesis‐related diseases. Table 1 exhibits the effect of compounds regulating Ca^2+^/CaN/NFAT signalling pathway on the differentiation of osteoblasts.

**TABLE 1:** The impact of Ca^2+^/CaN/NFAT signalling pathway modulating compounds on osteoblasts

Targets	Compounds	Osteoblastogenesis			Ca^2+^/CaN/NFAT signalling pathway			Reference
		Concentration	Cell type	Differentiation	Concentration	Cell type	Impact	
Ca^2+^	KMUP−1	5–10 μM	BMSCs/MC3T3‐E1	↑	10 μM	RAW264.7	↓	96, 97
	Zinc	1–50 μM	BMSCs/MC3T3‐E1	↓	10–30 μM	BMMs	↓	98, 99, 100, 101
	Cyanidin	50–200 μM	MC3T3‐E1	↑	80–300 μM 1–100 μg/ml 10 μM 5–10 μM	Pancreatic β cells PC12 cells C2C12 myoblasts RAW264.7	↑ ↑ ↑ ↓	102, 103, 104, 105, 106, 107, 108, 109
	Harpagoside	0.032–4 μM	MC3T3‐E1	↑	100 μM	BMMs	↓	110, 111, 112
	Artesunate	2.5–10 μM	BMSCs	↑	1.5–2.0 μM 15 µg/ml 16–32 μM 12.5 μM	ASMCs Erythrocytes HUVECs RAW264.7	↑ ↑ ↑ ↓	113, 114, 115, 116, 117
	Apocynin	0.1–1 μM	MC3T3‐E1	↑	1 μM	BMMs	↓	118, 119
	Amyloid β peptide	0.5–10 μM	MC3T3‐E1	↑	1–5 μM	BMMs	↑	120, 121, 122
CaM	KN−93	2 mM 10 μM	C2C12 cells BMSCs	↓ ↓	2 mM 10 μM	C2C12 cells BMSCs	↓ ↓	123, 124
	Trifluoperazine	10 μM	Calvarial model of mouse pups	↓	10 μM	Calvarial model of mouse pups	↓	83, 84
CaN	CsA	<1 μM >1 μM	BMSCs/MC3T3‐E1	↑ ↓	<1 μM >1 μM	BMSCs/MC3T3‐E1	↓ ↓	86, 87
	FK506	<1 μM >1 μM	BMSCs/MC3T3‐E1	↑ ↓	<1 μM >1 μM	BMSCs/MC3T3‐E1	↑ ↓	45, 88

### 4.1 KMUP‐1

Xanthine derivative KMUP‐1 (7‐[2‐[4‐(2‐chlorophenyl)piperazinyl]ethyl]‐1,3‐dimethylxanthine) can inhibit phosphodiesterase (PDE) activity, Liou et al. found that 5–10 μM KMUP‐1 can induce osteogenic differentiation of BMSCs and MC3T3‐E1 cells and promote mineralization.^96^ For the time being, there is no research showing the effect of KMUP‐1 on Ca^2+^ signal in BMSCs, MC3T3‐E1 cells or osteoblasts, but Liou et al.^97^ detected that 10 μM KMUP‐1 in RAW264.7 cells suppressed the RANKL‐induced Ca^2+^ oscillation and Ca^2+^ signal activation.

### 4.2 Zinc

Zinc is essential in the process of skeletal development, 1–50 μM zinc has been shown to inhibit osteoblast apoptosis and promote the proliferation and differentiation of osteoblasts,^98^ and adding zinc (25–200 mg/dl) to the cultured chicken embryo tibia has been demonstrated to lead to a concentration‐dependent increase in tibial ALP activity and an increase in the level of bone formation,^99^ physiological concentrations of zinc (25–200 mg/dl) have also been shown to increase bone resorption in tibia of chicken embryos.^100^ Similarly, the effect of zinc on Ca^2+^ in osteoblast‐related cells has not been exhibited, but it has been demonstrated that 10–30 μM zinc inhibited the increase in Ca^2+^ concentration in BMMs induced by RANKL, and 30–100 μm zinc inhibited the CaN activity of BMMs.^101^

### 4.3 Cyanidin

Cyanidin found in fruits and vegetables is a natural anthocyanin. Some previous studies have found that 50–200 μM cyanidin accelerated the proliferation, osteogenic differentiation and mineralization of MC3T3‐E1 cells.^102^, ^103^, ^104^ In rat pancreatic β cells, 80–300 μM cyanidin activates type I voltage‐dependent Ca^2+^ channel (VDCC) to promote Ca2+ influx, thereby increasing the intracellular Ca^2+^ concentration, the intracellular Ca^2+^ level increased the highest level when treated with 100 μM cyanidin.^105^ Similarly, 1–100 μg/ml cyanidin activates P2Y receptor‐mediated PLC in rat pheochromocytoma (PC12) cells and causes Ca^2+^ influx,^106^ Toshiya et al. claimed that 10 μM cyanidin could increase the level of intracellular cAMP by inhibiting PDE activity of the mouse C2C12 myoblasts, thereby promoting the elevation of intracellular Ca^2+^ concentration.^107^ However, in colon carcinoma cells, cyanidin inhibited the increase in intracellular Ca^2+^ level caused by neurotensin,^108^ and cyanidin at concentrations of 5–10 μM reduced the increase in intracellular Ca^2+^ concentration of RAW264.7 cells induced by RANKL.^109^

### 4.4 Harpagoside

Harpagoside is an iridoid glycoside extracted from harpagophytum procumbens var. sublobatum. Harpagide at concentrations of 0.032–4 μM promotes the osteogenic differentiation and mineralization of MC3T3‐E1 cells in a concentration‐dependent manner.^110^, ^111^ Kim et al.^112^ found that 100 μM Harpagoside inhibits the activation of Syk, Btk and PLCγ2 induced by RANKL in BMMs, further attenuates intracellular Ca^2+^ oscillations and reduces Ca^2+^ level.

### 4.5 Artesunate

Artesunate (ART) is a derivative of artemisinin, which has anti‐viral, anti‐tumour and anti‐malaria functions. Zeng et al.^113^ observed that 2.5–10 μM ART inhibited the expression of DKK1 in hBMSCs and increased the protein levels of cyclin D1, β‐catenin and c‐myc in a dose‐dependent manner, thereby promoting the process of osteogenic differentiation. Zeng et al.^114^ proved that 12.5 μM ART inhibited the activation of PLCγ1 and the increase of Ca^2+^ level induced by LPS in RAW264.7 cells, and also reduced the protein expression of the catalytic subunit of CaN. However, it is also reported that in airway smooth muscle cells (ASMCs), 1.5 and 2.0 mM ART significantly increased the intracellular Ca^2+^ concentration,^115^ Alzoubi et al.^116^ also reported that the treatment of 15 µg/ml ART can significantly increase the intracellular Ca^2+^ level of erythrocytes, Wu et al.^117^ found that human umbilical vein endothelial cells (HUVECs) cultured in Hanks solution containing Ca^2+^ rapidly increased intracellular Ca^2+^ concentration under the treatment of 16–32 μM ART.

### 4.6 Apocynin

The inhibitor of NADPH oxidase, apocynin, is a kind of methoxy‐substituted catechol. When MC3T3‐E1 cells are exposed to antimycin A and resulting in excessive ROS production, 0.01–1 μM apocynin can scavenge ROS, protect MC3T3‐E1 cells and promote their osteogenic differentiation.^118^ In BMMs, apocynin reduces Ca^2+^ influx by blocking Ca^2+^ channels except the two pore channel 2 (TPC2) and inositol 1,4,5‐triphosphate receptor 1 (IP3R1), causing reduction of intracellular Ca^2+^ concentration.^119^

### 4.7 Amyloid β peptide

Alzheimer's disease is characterized by the loss of synapses and neurons in the elderly, and the accumulation of amyloid β peptide (Aβ) is its hallmark. Research by Yang et al.^120^ showed that 0.5–10 μM Aβ can activate the Wnt signalling pathway by binding to LRP5/6 in MC3T3‐E1 cells, thereby promoting osteogenic differentiation. Aβ induces synaptic dysfunction by activating N‐methyl‐D‐aspartate receptors (NMDARs) to increase intracellular Ca^2+^ levels and activate related downstream signals.^121^ Besides, Li et al.^122^ also found that 1–5 μM Aβ increased intracellular Ca^2+^ levels and activated the Ca^2+^ signalling pathway during the process of inducing osteoclast differentiation and bone resorption in BMMs.

### 4.8 KN‐93

In osteoblast precursor cells, after Ca^2+^ binds to CaM, CaM activates a variety of target proteins, including calmodulin‐dependent protein kinase (CaMK). Choi et al. demonstrated that CaMKII participated in osteogenic differentiation of C2C12 mouse pre‐myoblast cell line induced by BMP‐4, and in the process of osteoblastogenesis, KN‐93 (2 mM), the inhibitor of CaMKII, blocked the osteogenic differentiation process of C2C12 cells induced by BMP‐4.^123^ Similarly, the research by Shin et al.^124^ reported that 10 μM KN‐93 inhibited the osteogenic differentiation and mineral deposition of hMSCs.

### 4.9 Trifluoperazine

Trifluoperazine (TFP) can inhibit the activity of CaM and further restrain the activation of CaMKII. 10 μM TFP inhibits osteogenic differentiation, and also shows the ability to reduce the formation and mineralization of osteoblasts in the calvarial model of mouse pups.^83^ Komoda et al.^84^ also confirmed the inhibitory effect of TFP on ALP activity in rat calvaria and its inhibitory effect on the proliferation and osteogenic differentiation of MC3T3‐E1 cells in vitro.

### 4.10 Cyclosporin A and FK506

Cyclosporin A and FK506 are CaN inhibitors and are widely used to reduce rejection reaction after organ transplantation. Low concentrations of CsA (less than 1 μM in vitro and 35.5 nM in vivo) have been shown to increase the expression of Fra‐2 to promote the transcription of osteogenic genes, thereby promoting osteogenic differentiation.^86^, ^87^ High concentrations of CsA (more than 1 μM in vitro and in vivo) inhibit osteogenic differentiation and bone formation.^86^ Similarly, low concentrations of FK506 (less than 1 μM in vitro) promote osteogenic differentiation,^88^ whilst high concentrations of FK506 can also reduce BMP‐2 induced osteogenic differentiation both in vivo and in vitro.^45^ The osteoinhibitory effect of high concentration of CsA and FK506 is believed to be exerted by inhibiting the formation of NFAT‐Osterix‐DNA complex.

## 5 SUMMARY AND OUTLOOK

A variety of existing evidences indicate that the Ca^2+/^CaN/NFAT signalling pathway, which is an extremely important part of growth and development, is inextricably linked to bone formation. The regulation of each components in the signal pathway, such as activation, inhibition, overexpression, silencing, etc., often resulting in changes in the process of osteoblast differentiation in vivo and in vitro. In the process of osteoblast differentiation, the non‐canonical Wnt pathway triggers the activation of Ca^2+^/CaN/NFAT signalling pathway, then NFAT and Osterix form transcription complexes to induce the expression of downstream osteogenic‐related genes. Some research teams studied the effect of CaN on osteogenic differentiation by means of gene deletion or gain‐of‐function but came to diametrically opposite conclusions. The most important differences in the experimental methods of these teams lie in the different CaN subunits they target, and whether the intervention is limited to osteoblasts. In addition, the osteogenic function of NFAT has also been questioned, some studies claimed that it acted as a transcription factor to promote the expression of osteoblast‐related genes, whilst some other studies believed that NFAT inhibited the differentiation of osteoblasts by inhibiting Fra‐2. Most researchers agreed that low‐concentration CaN inhibitors promoted osteogenic differentiation, and high‐concentration CaN inhibitors suppressed the process of osteogenic differentiation, but no specific limit of the concentration of CaN inhibitor and the actual mechanism were given to explain the reason. In connection with the study of CaN inhibitors in osteoclasts, we speculate that immunophilin should also be included in the analysis of its influence on osteogenic differentiation.

A variety of existing compounds have the ability to promote or inhibit osteogenic differentiation, whilst regulating the Ca^2+^/CaN/NFAT signalling pathway. However, we have found that some compounds positively regulate Ca^2+^ signal and promote osteogenic differentiation, whilst some compounds negatively regulate Ca^2+^ signal and promotes osteogenic differentiation as well. The reason for this paradox may lie in the different types of cells used in these studies for Ca^2+^ signal and the ability to regulate osteogenic differentiation, and the different application concentrations of the compounds, or the signalling pathway involved in the compounds driving osteogenic differentiation is not Ca^2+^/CaN/NFAT but other signalling pathways. The information we have collected and summarized can be used to investigate the relationship between the Ca^2+^/CaN/NFAT signalling pathway and osteogenic differentiation as well as providing some ideas for exploring better treatment options for regulating bone formation‐related diseases, and these remaining uncertain mechanisms require further research.

Over the years, the relationship between Ca^2+^/CaN/NFAT signalling pathway and bone metabolism has been explored in many ways, this signalling pathway has a wide range of effects on cell fate, and the mechanisms involved are far‐reaching. There are still many unknown or unexplained relationships between Ca^2+^/CaN/NFAT signalling pathway and osteoblastogenesis, further exploration in this field is needed to broaden the way for the study of bone formation regulation and bone‐related diseases development.

## CONFLICT OF INTERESTS

The authors declare that they have no conflict of interest.

## AUTHOR CONTRIBUTIONS

W.X. and R.R. conceived the aims and structure of the review. R.R. and J.G. collected the articles and wrote the original draft of the manuscript. Y.C., Y.Z. and L.C. reviewed and edited the manuscript. W.X. acquired the funding. All authors have read and agreed to the published version of the manuscript.

## Data Availability

All the data are available from the corresponding author by request.
